# Transfusion

**Published:** 1854-03

**Authors:** John Soden


### ECLECTIC DEPARTMENT.

### *Case of Hæmorrhage from the Uterus in which transfusion was successfully performed, with remarks on transfusion generally.*

BY JOHN SODEN, F. R. C. S.

I am impressed with a strong belief, that the extent of the evidence in favor of transfusion is very little known to the profession at large; that false notions prevail respecting its dangerous character, and the difficulties attending upon its execution; and that especially it does not receive justice at the hands of modern authorities in the practice of midwifery. My interest was first engaged in the subject by an opportunity which occurred to me three years ago, of proving its power in the case which forms the subject of this paper.

Case.—On January 7th, 1849, a lady was attended in Bath by Mr. Ormond, a gentleman of great experience and intelligence, at the birth of her second child. The labor was rapid, and the latter pains so severe that the uterus was violently emptied of its contents and became inverted; a gush of blood ensued, and the patient fainted—the placenta was detached and the uterus returned to its natural position. No further hæmorrhage took place—I joined Mr. Ormond in about half an hour—the patient had not rallied—she was lying on her back, insensible, perfectly cold, pulseless, and exsanguine in appearance—the only signs of life were short, jerking respirations at long intervals, and of a stertorous character. Nothing could look more unpromising than her condition. Mr. Ormond was using means to restore warmth by friction, mustard poultices, &c., and was administering brandy by teaspoonfuls, which the patient was just able to swallow. These means had now been employed for more than half an hour without any effect, and there scarcely seemed any hope that the patient would recover from the extreme exhaustion in which she then lay. We agreed that transfusion was the only resource, and I left Mr. Ormond to procure the instrument that occurred to me at the moment as best adapted for the purpose. I returned in half an hour, and during the interval Mr. Norman had arrived. No change had taken place in the condition of the patient; and as we were now prepared to act, we determined to wait a little longer to watch for an indication of the course the case would take. This was presently afforded in an unmistakeable manner. The patient was no longer able to swallow, the respirations became more rare and stertorous, and were evidently on the point of ceasing altogether. Transfusion was now had recourse to, and the following plan was adopted for its execution. My instrument was a well made syringe of German silver with a detached stop-cock; it was larger than desirable, being capable of holding seven ounces. Mr. Norman exposed the external cephalic vein, by an incision two inches in length, (the arm being fat.) Mr. Ormond bled the husband, the blood being received into a small deep basin, standing in another containing hot water. As soon as sufficient blood had been drawn, I filled the syringe, previously well warmed and invested with a hot cloth, and at once proceeded to inject the blood into the vein. At first it would not pass up, but returned by the side of the pipe; presently the opposition from the close contact of the coats of the vein seemed to give way, and the blood, though impelled by a steady and moderate pressure, rushed up the vein with a rapidity that the eye could scarcely trace. The effect was electrical; instantaneously a convulsion seized the whole frame, and the muscles of the face were frightfully distorted. I paused in the injection, and I do not think more than an ounce could have found its way into the circulation; happily it was sufficient: the convulsion was but momemtary, and signs of returning animation immediately succeeded. A restless movement pervaded the whole body—the arms were tossed over the head, and although consciousness did not return, the patient faintly but audibly spoke, muttering two or three times the expression "so tired," "so tired," she seemed to pass from a state of coma into one of syncope. The heart's action was now distinctly perceptible, and the vital energy gradually but very slowly returned: it was full an hour before any pulse could be felt at the wrists, and though the recovery steadily progressed without relapse, the patient did not recover consciousness until the following morning. During the whole of this time, every means was used to promote warmth, and no difficulty was experienced in getting the stimulants swallowed, that were from time to time administered. Some inflammation was set up in the fore arm below the point of the incision, but it was not of any moment, and subsided in two or three days, with the application of fomentations only. I hear from Mr. Ormond, that the patient remained for a long time in an exsanguine state, and complained of weakness and pain in the back. She did not nurse her child, and the catemenia returned in three months. After this period she left for change of air, and Mr. Ormond lost sight of her, but he subsequently heard, that, suffering from leucorrhœa, she went to London, and was treated for ulceration of the womb. This lady is now in India; recent accounts have been received of the birth of another child and the well-doing of the mother.

The details of this case exemplify most of the important points connected with the performance of the operation. The rapid and decisive results obtained are by no means singular; on the contrary, the narrators of many cases show parallel success, and describe in forcible language the wonder and surprise excited in their minds by the almost miraculous resuscitations they had witnessed. An analysis shows that out of thirty-six cases, twenty-nine recovered from imminent death by the operation of transfusion, and in seven only its performance was unsuccessful in restoring animation. It does not appear that the fatal termination in any case was due to or hastened by the operation, though in two instances, the latter effect was, on no warrantable grounds, attributed to its influence.

[An accompanying table of thirty-six cases, is omitted for want of room.]

Transfusion is not to be regarded simply as the restoration of a certain deficiency of blood ; for the benefit it affords bears but a trifling relation to any rule of loss and supply; nor is its agency to be attributed solely to its mechanical influence, as a warm fluid upon the heart. The transfusion of human blood may claim to be a direct, powerful,—I might almost add, when applied early,—unfailing stimulus to exhausted energy, even at the lowest point of existence, and when past the restorative aid of any other known means, either extraneous or inherent. The rapidity of the revival, however, varies; it depends not only on the circumstances of the case, as regards the previous duration and cause of the exhaustion, which exerts a material influence upon the power of the remedy, but also on the character of the means used. Under this head, I include the quantity of the fluid to be transfused, and the mode of operation. Inattention to these three points, was, perhaps, the cause of failure in five of the seven unsuccessful cases.

From the table it appears that the total *quantity* transfused, varied exceedingly; the lowest amount named is from one to two ounces; the highest, twenty-four ounces and a-half. The intermediate quantities varying a good deal, but with a preponderance to the low amount.

| | | | | | |
|---|---|---|---|---|---|
| 1 oz. | was injected in | 1 case, | 10 oz. | were injected in | 4 cases, |
| 2 oz. | " | 2 cases, | 12 oz. | " | 1 case, |
| 2½ oz. | " | 3 cases, | 14 oz. | " | 2 cases, |
| 4 oz. | " | 7 cases, | 15 oz. | " | 2 oases, |
| 5 oz. | " | 1 case, | 16 oz. | " | 1 case, |
| 6 oz. | " | 2 cases, | 22 oz. | " | 2 cases, |
| 8 oz. | " | 1 case, | 24½ oz. | " | 1 case, |
| 9 oz. | " | 2 cases, | Quantity not named in 4 cases.* | | |

No mean can be taken from this data to afford a practical guide—the quantity required was determined by the peculiar circumstances of each case; but generally, it may be said that the lesser amount was needed in proportion as the exhaustion arose from the suddenness of the shock rather than from the extent of the hæmorrhage before labor. In case 36, the heat responded immediately to the application of the stimulus. In case 24, where the hæmorrhage had been violent, and the coma complete, no pulse having been perceptible in the carotids for two hours and a half, as much as twelve ounces was injected without any effect being produced, but recovery gradually progressed as the injection was proceeded with. It does not appear that too much blood was ever injected, or that any signs presented themselves indicating repletion. A large quantity of fluid may be introduced into the system without effect. In cholera, more than one hundred ounces of saline injection were sometimes used. Among the

* The quantity employed in each of the unsuccessful cases, in this table, was as follows: in two, 4 oz.; in two, 10 oz; in one, 14 oz.; in one 16 oz.; and in one, unknown.

prejudices existing against transfusion, the fear of serious consequences from engorgement of the heart or brain, by the injection of too large a quantity, has been injuriously influential. On some occasions the injection was not pushed to the extent of producing any effect; in such cases its power was not tested at all, and life was probably sacrificed by the over-caution of the operator. The evidence regarding the *quality* transfused is very conclusive as to its importance. The propriety of selecting a good subject to supply the blood has been anticipated, and strongly inculcated by the most of the advocates of transfusion; but experience has shown that this is of more consequence than could have been expected. In the early days of transfusion the blood of animals was frequently employed; and even recently the blood of a goat was successfully transfused in a case of hæmorrhage from phthisis. With the more accurate knowledge which we now possess of the physical distinctions between human blood and that of animals, such an example, though it was successful, is not likely to be followed, and the objections to it are too obvious to need recital. When transfusion was expected to work the miracle of perpetuating youth and curing all diseases, a variety of substances, such as gum, camphor, &c., was introduced into the veins; the failure of these experiments soon brought the remedy into disfavor. The idea, however, of the transfusion of artificial substances is not altogether to be condemned. It appears that the blood transfused may be drawn from different individuals with equally good effect, as from one alone, and that no evil results from the combination.

Regarding the *mode of performing* transfusion, I have come to the usual conclusion, that the most simple means are the best. No instrument is so convenient or safe for the purpose, as a common syringe. It should be accurately made, fitted with a detached stop-cock, plated or tinned on the inside, and capable of holding about three ounces. An infinite variety of apparatus has been invented for transfusing, not so much with the object of facilitating its performance, as of guarding against the danger of admitting air with the fluid injected: a danger that is nearly imaginary, and not founded on any ground of probability. An impression of this danger so generally exists in the profession, that it has been the greatest obstacle to the more frequent employment of transfusion. In many of the cases cited, the instrument used is not mentioned; sometimes a special apparatus was employed, and often a common syringe. No difficulty appears to have been experienced from the nature of the instrument used, except in one instance, and in that case the special apparatus was changed for a simple syringe. Dr. Marmonier is said to have used a child's common toy syringe; and great credit is due to him, that with such simple means, and without assistance, he undertook and successfully performed the operation. In the account of another case, I have stated the impediment the empty state of the vein seemed to offer to the entrance of the blood, and that on the opposition being overcome the wave of blood was traced with great rapidity towards the heart. It is well known that the danger of air finding admission into the veins is greater in operations about the neck than in those upon the extremities. I do not imagine that this circumstance depends upon the greater size of the veins, or their contiguity to the heart, as in the former case, but on the mechanical cause of the different incidence of the atmospheric pressure. A vein when divided remains empty for a considerable distance above the point of division, and the air is prevented from entering by its own external pressure along the trunk; by this means the coats of the vein are maintained in contact. In the neck this does not so well apply; the external pressure can there only fall upon the orifice, when the division is low down, and not upon the trunk beyond; consequently, there is less opposition to the suction of the air into the vacuum of the vein: on this account a vein in the neck ought never to be chosen for the operation. In all the cases in the list, the usual situation of venesection was adopted, except in one, where the internal jugular was selected, from there being no apparent vein in the arm; the patient died in an hour after the transfusion. As the presence of air was on a post-mortem examination, demonstrated in the heart, a doubt existed whether it was attributable to the injection or to decomposition. No circumstances occurred at the moment of transfusion to indicate that the accident had happened; and where it has taken place in the division of veins during an operation, it has always declared itself,—sometimes by an audible sound with which the air rushes into the vacuum. In performing transfusion there is but one source of danger from this circumstance, and strangely enough, though it is the most obvious, it has been entirely overlooked by the different inventors of special apparatus. Many of their instruments are ingeniously calculated to promote the very object they are designed to prevent, namely, the passage of air with the blood through the instrument, and none of them offer an equal security, on this point, to a common syringe when used with proper and obvious precautions. The syringe has also the same advantage against the only chance through which the air might gain admission into the vein, in a dangerous form, for a few bubbles injected with the blood are unimportant, and that is, its passage, by the side of the instrument into the empty vein, when the coats of the latter are separated by the introduction of the pipe; pressure beyond the opening till the blood is emerging from the syringe would effectually guard against this possibility, or by the injection being performed under water.

In conclusion I will briefly remark on the general applicability of this remedy. An equal success has attended its adoption in the exhaustion caused simply by loss of blood under any circumstances. It has been chiefly tried after wounds and operations, of which Dr. Routh gives many examples. Its power is equally great where the blood diminished in the system depends upon inanition, as upon direct loss. A very valuable case illustrating this point has been published by the late Dr. Prichard and Mr. Clarke. The case was one of atrophy from dyspepsia and constant vomiting. Sixteen oz. were injected, and the patient instantly revived, and completely recovered in three months. Transfusion has been of temporary service even where organic disease has been present, as phthisis and cancer of the stomach. Where an animal poison exists in the system in an active form, it has not been successful, as in hydrophobia, where it failed in the hands of Dieffenbach and Magendie; but this is an untried field. Dr. Routh especially alludes to the benefit that might be expected from its employment in the collapse of typhus fever. I witnessed its trial in a case of this description, several years ago, by Dr. Stokes, at the Meath Hospital. The patient was a middle-aged woman. Eight or ten ounces were injected; a faint and but momentary indication of rallying was induced. The injection was not repeated, and the woman died in three days afterwards.

Dr. Routh also suggests its use in the diarrhœa of children, where fatal exhaustion is threatened. There are most promising indications for its employment under this head. The numbers of children who die from defective nutrition, arising from imperfect power of assimilation,—their aspect years behind the standard of their age,—who drag on, by aid of iron, iodine, and now of cod liver oil, a miserable existence, to be at last prematurely terminated,—in such cases it is not irrational to anticipate that transfusion, when fairly tested, may prove a remedial agent of greater power and efficacy than any we now possess.—*Med. Chirurg. Trans.*

